# The Impact of Periodontal Disease on Hospital Admission and Mortality During COVID-19 Pandemic

**DOI:** 10.3389/fmed.2020.604980

**Published:** 2020-11-23

**Authors:** Harriet Larvin, Sheryl Wilmott, Jianhua Wu, Jing Kang

**Affiliations:** ^1^School of Dentistry, University of Leeds, Leeds, United Kingdom; ^2^Leeds Dental Institute, Leeds Teaching Hospitals Trust, Leeds, United Kingdom; ^3^Leeds Institute for Data Analytics, University of Leeds, Leeds, United Kingdom; ^4^Oral Biology, School of Dentistry, University of Leeds, Leeds, United Kingdom

**Keywords:** COVID-19, oral health, periodontal disease, hospital admission, mortality, UK Biobank

## Abstract

**Introduction:** COVID-19 has had a huge impact on society and healthcare and it has been suggested that people with periodontal disease are at risk of having worse outcomes from the disease. The aim of this study was to quantify the impact of periodontal disease on hospital admission and mortality during the COVID-19 pandemic.

**Materials and Methods:** The study extracted UK Biobank participants who had taken a COVID-19 test between March and June 2020 (*n* = 13,253), of which 1,616 were COVID-19 positive (12%) and 11,637 were COVID-19 negative (88%). Self-reported oral health indicators of painful or bleeding gums and loose teeth were used as surrogates for periodontal disease, participants who did not report any of the aforementioned indicators were used as controls. Multivariable logistic regressions were used to obtain crude and adjusted odds ratios of COVID-19 infection, subsequent hospital admission and mortality adjusted for demographics, BMI, biomarkers, lifestyle and co-morbidities.

**Results:** Painful gums, bleeding gums and loose teeth were reported in 2.7, 11.2 and 3.3% of participants with COVID-19 infection, respectively. Risk of COVID-19 infection in participants with painful or bleeding gums and loose teeth compared to controls was not increased (odds ratio [OR]: 1.10, 95% CI: 0.72–1.69; OR: 1.15, 95% CI: 0.84–1.59). COVID-19 positive participants with painful or bleeding gums had a higher risk of mortality (OR: 1.71, 95% CI: 1.05–2.72) but not hospital admission (OR: 0.90, 95% CI: 0.59–1.37). Participants with loose teeth did not show higher risk of hospital admission or mortality compared to the control group (OR = 1.55, 95% CI: 0.87–2.77; OR: 1.85; 95% CI: 0.92–2.72).

**Conclusion:** There was insufficient evidence to link periodontal disease with an increased risk of COVID-19 infection. However, amongst the COVID-19 positive, there was significantly higher mortality for participants with periodontal disease. Utilization of linked dental and hospital patient records would improve the understanding of the impact of periodontal disease on COVID-19 related outcomes.

## Introduction

The COVID-19 pandemic has resulted in a huge burden on society and healthcare. Studies have shown that sex, older age and co-morbidities including diabetes, hypertension, cardiovascular disease and cancer may increase risk of COVID-19 associated deaths (1, 2). There are also suggestions that COVID-19 related deaths may be associated with deprivation and ethnicity (3). As yet no study has examined the impact of periodontal disease on COVID-19 infection and associated outcomes.

Over a lifetime, ~90% of adults will experience oral disease. Periodontal disease is a main cause of tooth loss in adults and the sixth most prevalent disease globally (4), affecting between 10 and 50% of adults (5, 6). Existing evidence suggests that people with periodontitis may have an increased risk in developing subsequent systemic diseases including cardiovascular disease, hypertension, respiratory disease, diabetes and cancer (7–12). It has been suggested that COVID-19 could be linked to periodontal disease given their shared risk factors, which include obesity, age and hypertension (13, 14). Additionally, there is growing evidence of bacterial co-infection in COVID-19 hospitalizations (15), while ventilator associated pneumonia is also a reported complication of patients hospitalized with COVID-19 (16). Oral dysbiosis as a result of increased dental plaque in periodontitis may provide an environment for oral carriage of respiratory pathogens and cause COVID-19 complications. As yet there is insufficient evidence to provide robust conclusions on how periodontal disease may be associated to COVID-19 infection and outcomes.

During the COVID-19 pandemic UK Biobank has provided live-feed information of its participants on COVID-19 testing, subsequent hospital admission and mortality. The UK Biobank dataset provides an extensive resource to aid in understanding the impact of various factors including periodontal disease during the pandemic. The aim of this study was to quantify the impact of periodontal disease on COVID-19 infection and related outcomes utilizing the UK Biobank data.

## Materials and Methods

### Database

Data from the UK Biobank cohort was used in this analysis. UK Biobank is a national, longitudinal cohort study of over 500,000 participants. The dataset includes information from UK Biobank assessment centers; self-reported responses *via* online questionnaires, linked HES data including ICD-10 diagnoses and hospital admissions provided by NHS Digital, and death records extracted from the death register. Since 16th March 2020 COVID-19 test results provided by Public Health England have been linked to UK Biobank core data as a live-feed for the purposes of COVID-19 related research during the pandemic. The COVID-19 test result data reflects the change in national testing capacity, which has moved from mostly inpatient to more community testing. The data is accessible by researchers with an approved UK Biobank application (reference no: 54633) for a prior study and may carry out additional COVID-19 related research without further approval according to UK Biobank guidelines.

Study recruitment was between 2006 and 2010. Linked follow-up information from HES and death records were available until May and June 2020, respectively. COVID-19 test results were available until the date of data extraction (August 2020). All participant information was fully anonymised prior to data acquisition. HES data, death records and COVID-19 test results were linked to the core UK Biobank dataset by a unique study ID therefore we do not anticipate any missing outcome data. Participants are free to remove themselves from the UK Biobank study cohort at any point (17).

### Study Sample

For this study we included participants who were tested for COVID-19 (*n* = 13,502) with their results linked to the core UK Biobank dataset. The final dataset included information on participant demographics, biomarkers, co-morbidities, COVID-19 test results, hospital admissions and mortality.

### Study Outcomes

Hospital admission and mortality following positive COVID-19 test result.

### Exposures

The self-reported oral health indicators of bleeding gums, painful gums and loose teeth were utilized as surrogates for periodontal disease as they have demonstrated their validity in the absence of a clinical diagnosis (18). Painful and bleeding gums were associated with mild to moderate periodontal disease, while loose teeth indicated severe periodontal disease. Periodontal disease status was determined by the presence of any of the aforementioned indicators, while no mention of the indicators identified the control group, comprising participants with no self-reported history of periodontal disease.

### Covariates

Information on participant demographics (age, sex, ethnicity, household income) and BMI was collated during attendance to UK Biobank assessment centers. Periodontal disease status was also determined from self-reported responses taken from UK Biobank assessment centers. Age at COVID-19 test was derived from age at assessment center attendance and date of test. Information on biomarkers such as blood pressure (systolic and diastolic) and resting heart rate was also acquired from attending UK Biobank assessment centers. Where there were multiple entries for biomarkers the most recent report was extracted. History of smoking was derived according to self-reported current or ex-smoking status. The following conditions were also considered as covariates: cancer, hypertension, angina, cardiac arrest, diabetes, myocardial infarction (MI), stroke, peripheral artery disease (PAD), heart failure, atrial fibrillation and respiratory conditions. Validated ICD-10 code lists within the Cardiovascular Research Using Linked Bespoke Studies and Electronic Health Records (CALIBER) resource were used to classify the aforementioned conditions (19), and the presence of the appropriate ICD-10 code in a participants health records denoted history of the disease. The code list meanings were also adapted to identify relevant self-reported conditions that were not coded with ICD-10 classification. Participants were considered to have disease history if relevant ICD-10 codes were found in their health records, or if the condition was self-reported at the assessment center. No participants had history of heart failure therefore it was excluded from further analysis. A history of hypertension was determined if ICD-10 code was present in health record data, or if the blood pressure reading from the assessment center exceeded 140/90 mmHg.

### Data Analysis

Descriptive statistics for baseline characteristics were presented using frequency (percentage) for categorical data and mean [standard deviation (SD)] or median [interquartile range (IQR)] for continuous variables depending on their distribution. Among participants who had COVID 19 test results available, the risk of COVID-19 infection was investigated for those with periodontal disease (self-reported painful gums, bleeding gums or loose teeth) using logistic regression, and further adjusted for covariates including demographics, BMI, biomarkers, lifestyles and comorbidities. Among participants with a positive COVID-19 test result, the risks of hospital admission and mortality were investigated for those with periodontal disease using logistic regression and further adjusted for the relevant covariates mentioned above. Crude and adjusted odds ratios (OR) with 95% confidence interval were reported.

Multiple imputations were used for missing data, and Rubin's rule was used to combine the coefficients (20). To assess the impact of missing data, sensitivity analyses were performed using only the complete cases. Data processing and analyses were performed using R version 4.0.0 (21). Statistical significance level was set as 0.05.

## Results

Of the 13,253 participants involved in the final analysis cohort, there were 365 (2.4%) participants with painful gums, 1,329 (8.7%) with bleeding gums, 406 (2.7%) with loose teeth and 11,153 (84.1%) with no self-reported history of periodontal disease. Overall there were 1,616 (10.5%) confirmed COVID-19 cases and 11,637 negative test results in the study sample.

The mean age of all participants was 68.55 ± 8.38 years. There were 51.3% females in the final cohort. 92.9% of participants were of white ethnicity. On average participants were considered overweight with a mean BMI of 28.24 ± 5.18.Mean BMI was higher in COVID-19 positive participants when compared to negative participants across all periodontal disease indicator groups. 58.7% of all participants had a history of hypertension and 23.1% had respiratory disease (Table 1).

**Table 1:** Summary table of UK Biobank participants stratified by oral health indicator and COVID-19 test result.

		**No self-reported history of periodontal disease**		**Painful gums**		**Bleeding gums**		**Loose teeth**	
		**COVID-19 test result**		**COVID-19 test result**		**COVID-19 test result**		**COVID-19 test result**	
	**Overall**	**–**	**+**	**–**	**+**	**–**	**+**	**–**	**+**
*n*	13,253	9,815	1,338	321	44	1,148	181	353	53
Age at test, mean (SD)	68.55 (8.38)	69.10 (8.20)	67.15 (9.19)	67.88 (8.71)	69.27 (9.20)	66.10 (8.22)	63.24 (8.50)	69.82 (7.27)	68.13 (8.28)
Sex, female (%)	6,802 (51.3)	5,026 (51.2)	627 (46.9)	174 (54.2)	18 (40.9)	677 (59.0)	98 (54.1)	150 (42.5)	32 (60.4)
Ethnicity, white (%)	12,268 (92.9)	9,231 (94.3)	1,180 (88.5)	276 (86.5)	34 (77.3)	1,047 (91.4)	148 (82.2)	307 (87.2)	45 (84.9)
**Average Total Household Income, £ (%)**									
<18,000	3,349 (28.3)	411 (4.7)	34 (2.9)	114 (43.3)	11 (29.7)	263 (27.0)	40 (26.0)	126 (43.4)	21 (51.2)
18,000–30,999	2,836 (24.0)	2,108 (24.1)	279 (23.5)	59 (22.4)	13 (35.1)	256 (26.3)	43 (27.9)	73 (25.2)	5 (12.2)
31,000–51,999	2,578 (21.8)	1,925 (22.0)	258 (21.8)	47 (17.9)	6 (16.2)	235 (24.2)	42 (27.3)	54 (18.6)	11 (26.8)
52,000–100,000	1,871 (15.8)	1,424 (16.3)	180 (15.2)	36 (13.7)	5 (13.5)	171 (17.6)	21 (13.6)	30 (0.3)	4 (9.8)
>100,000	517 (4.4)	481 (5.5)	73 (6.2)	7 (2.7)	2 (5.4)	48 (4.9)	8 (5.2)	7 (2.4)	NA
BMI, mean (SD)	28.24 (5.18)	28.08 (5.06)	28.68 (5.28)	28.83 (6.18)	30.55 (5.13)	28.61 (5.52)	28.27 (4.94)	28.76 (5.42)	30.81 (7.21)
Systolic blood pressure/mmHg, mean (SD)	140.69 (20.11)	141.03 (20.01)	140.78 (20.65)	138.43 (19.33)	143.28 (20.34)	138.44 (20.11)	138.06 (19.09)	141.88 (21.25)	136.21 (19.39)
Diastolic blood pressure/mmHg, mean (SD)	82.27 (10.79)	82.16 (10.74)	82.73 (11.16)	81.42 (9.86)	83.49 (10.76)	82.55 (10.92)	82.65 (10.99)	83.18 (10.77)	81.08 (11.95)
Resting heart rate/BPM, mean (SD)	63.85 (9.87)	64.12 (10.29)	57.67 (5.20)	58.00 (NA)	NA	67.14 (8.91)	65.00 (NA)	74.50 (7.78)	NA
History of smoking (%)	6,731 (51.0)	4,937 (50.5)	677 (51.0)	186 (58.7)	25 (56.8)	538 (47.0)	82 (45.6)	256 (73.1)	30 (56.6)
Cancer (%)	2,334 (17.6)	1,813 (18.50)	179 (13.4)	53 (16.5)	2 (4.5)	190 (16.6)	23 (12.7)	66 (18.7)	8 (15.1)
Hypertension (%)	7,781 (58.7)	5,776 (58.8)	793 (59.3)	197 (61.4)	31 (70.5)	636 (55.4)	90 (49.7)	229 (64.9)	29 (54.7)
Angina (%)	1,711 (12.9)	1,251 (12.7)	159 (11.9)	64 (19.9)	13 (29.5)	135 (11.8)	20 (11.0)	61 (17.3)	8 (15.1)
Cardiac arrest (%)	204 (1.5)	160 (1.6)	20 (1.5)	6 (1.9)	NA	11 (1.0)	1 (0.6)	5 (1.4)	1 (1.9)
Diabetes (%)	551 (4.2)	376 (3.8)	62 (4.6)	26 (8.1)	3 (6.8)	36 (3.1)	10 (5.5)	31 (8.8)	7 (13.2)
Myocardial infarction (%)	1,133 (8.5)	830 (8.5)	114 (8.5)	56 (17.4)	5 (11.4)	74 (6.4)	14 (7.7)	37 (10.5)	3 (5.7)
Stroke (%)	769 (5.8)	547 (5.6)	92 (6.9)	35 (10.9)	5 (11.4)	55 (4.8)	8 (4.4)	22 (6.2)	5 (9.4)
Peripheral artery disease (%)	963 (7.3)	695 (7.1)	110 (8.2)	36 (11.2)	2 (4.5)	67 (5.8)	12 (6.6)	36 (10.2)	5 (9.4)
Atrial fibrillation (%)	1,100 (8.3)	826 (8.4)	116 (8.7)	34 (10.6)	5 (11.4)	74 (6.4)	16 (8.8)	30 (8.5)	1 (1.9)
Respiratory disease (%)	3,056 (23.1)	2,213 (22.5)	335 (25.0)	88 (27.4)	9 (20.5)	255 (22.2)	49 (27.1)	96 (27.2)	11 (20.8)
Hospital admission	4,083 (30.2%)	2,723 (27.7)	665 (49.7)	89 (27.7)	18. (40.9)	297 (25.9)	81 (44.8)	101 (28.6)	26 (49.1)
Mortality	644 (4.8%)	292 (3.0)	247 (18.5)	12 (3.7)	7 (15.9)	25 (2.2)	21 (11.6)	11 (3.1)	16 (30.2)

*BMI, body mass index; n, number of participants; SD, standard deviation; NA, not applicable as not enough data; –, negative COVID-19 test result; +, positive COVID-19 test result*.

*Means and percentages are calculated for variables excluding missing data. There was missing data in the following variables: oral health (1.88%), ethnicity (0.3%), household income (15.9%), BMI (3.0%), systolic and diastolic blood pressure readings (3.4%), heart rate (99.4%), history of smoking (0.4%)*.

COVID-19 infection risk was not higher in participants with painful/bleeding gums or loose teeth compared to controls (OR = 1.10, 95% CI = 0.72–1.69; OR = 1.15, 95% CI = 0.84–1.59) (Table 2).

**Table 2:** Association between oral health indicators and risk of COVID-19 infection.

	**Oral health status, OR (95% CI)**		
	**Control**	**Bleeding or painful gums**	**Loose teeth**
Crude OR (95% CI)	**1 (ref)**	1.07 (0.70-1.63)	0.99 (0.73-1.38)
Adjusted^*^ OR (95% CI)	**1 (ref)**	1.10 (0.72-1.69)	1.15 (0.84-1.59)

*CI, confidence interval; OR, odds ratio; ref, reference value*.

* *Adjusted by age at test, sex, ethnicity, average total household income, BMI, systolic and diastolic blood pressure, history of smoking, history of previous conditions including: cancer, hypertension, angina, cardiac arrest, diabetes, myocardial infarction, stroke, peripheral artery disease, atrial fibrillation, and respiratory disease*.

Though participants with painful/bleeding gums did not have a higher risk of hospital admission (OR = 0.90, 95% CI = 0.59–1.37), their mortality was almost doubled (OR = 1.71, 95% CI = 1.05–2.72) compared to the control group. Participants with loose teeth did not have significantly increased risk of hospital admission or mortality (OR = 1.55, 95% CI = 0.87–2.77; OR = 1.85; 95% CI = 0.92–2.72) (Table 3).

**Table 3:** Association between oral health indicators and hospital admission and mortality for participants with COVID-19 infection.

	**Crude OR (95% CI) for oral health status**			Adjusted^*^ **OR (95% CI) for oral health status**		
	**Control**	**Painful or bleeding gums**	**Loose teeth**	**Control**	**Painful or bleeding gums**	**Loose teeth**
Hospital admission	**1 (ref)**	0.99 (0.13–3.02)	0.88 (0.16–10.04)	**1 (ref)**	0.91 (0.12–2.94)	0.90 (0.16–10.63)
Mortality	**1 (ref)**	1.60 (1.03–2.42)	1.34 (0.71–2.76)	**1 (ref)**	1.71 (1.05–2.72)	1.85 (0.92–2.72)

*CI, confidence interval; OR, odds ratio; ref, reference value*.

* *Adjusted by age at test, sex, ethnicity, average total household income, BMI, systolic and diastolic blood pressure, history of smoking, history of previous conditions including: cancer, hypertension, angina, cardiac arrest, diabetes, myocardial infarction, stroke, peripheral artery disease, atrial fibrillation, and respiratory disease*.

Sensitivity analysis on complete cases showed similar effects of periodontal disease on COVID-19 infection risk, hospital admission and mortality rate, but the effects were not significant due to a smaller sample size.

## Discussion

Our study did not find a difference in the risk of COVID-19 infection between participants with periodontal disease and those with no self-reported history of periodontal disease. However, logistic regression showed that participants with painful or bleeding gums were at much higher risk of mortality following COVID-19 infection after adjusting for covariates, though hospital admission risk was not higher in these participants. Loose teeth did not affect COVID-19 infection risk, or hospital admission and mortality following COVID-19 infection.

In this study, risk of mortality was significantly increased in participants with painful or bleeding gums following COVID-19 infection. A recent retrospective study of COVID-19 patients found dominant bacterial, viral and fungi co-infections in ~94% of cases (22). These pathogens have also been identified in the oral biofilms associated with periodontal disease (23). Our results suggest oral bacterial load in people with periodontal disease may influence prognosis following COVID-19 infection, and supports the suggestion that the oral microbiome could be associated with severe COVID-19 complications (13). Conversely, risk of hospital admission following COVID-19 infection in participants with painful or bleeding gums did not significantly increase. This could be due to a reduction in the number of people attending hospital for acute disease during the social containment mandate (“lockdown”) period of the pandemic (24). People with severe COVID-19 may have chosen to avoid hospital which may have impacted the risk estimates for this outcome. The follow up time for mortality outcomes from the UK Biobank cohort was longer than for the HES data available in determining hospital admission (June 2020 and May 2020, respectively). This may also influence the accuracy of estimates for risk of hospital admission.

Risk of hospital admission and mortality was not higher in participants with self-reported loose teeth, due to smaller sample size in this subgroup. Loose teeth is an indicator of severe periodontal disease (25). Participants with this response in the UK Biobank cohort could have since undergone periodontal treatment, had the affected teeth extracted, or in severe cases, the affected teeth may have self-exfoliated. This could result in a change in participant oral health status between the time of self-report and the COVID-19 infection as most of the self-reported information was collected at baseline. A study of a larger sample size with up-to-date dental measures is required to understand this phenomenon.

The results of this study have clinical implications for the management of COVID-19 infections in the general population, and in hospitalized patients. In England, routine dental services were suspended in March 2020 (26) and, at the time of writing, two-thirds of dental practices were operating at or below 25% of their pre-COVID-19 activity levels (27). Dental practices are also having difficulties sourcing Personal Protective Equipment (PPE) (28). In the absence of routine dental care, remote consultations are an opportunity to emphasize self-care methods such as thorough brushing and interdental cleaning, both to prevent oral disease and to reduce the risk of COVID-19 mortality. UK government strategy has focused on tackling obesity to reduce the risk of serious illness or death from COVID-19 (29). Our results show that periodontal disease may be another preventable risk factor that the government could target to improve population outcomes before and after COVID-19 infection. The oral health of patients who have been hospitalized with COVID-19 should not be neglected. There is a wealth of evidence establishing an association between good hospital mouth-care and a reduced risk of pneumonia and acute viral respiratory infections (13). Furthermore, simple measures such as chlorhexidine mouth rinses have been shown to reduce the risk of ventilator associated pneumonia in critically ill patients from 24 to 18% (30). Improving the mouth care provided to COVID-19 patients in hospital could be a straightforward method for improving their outcomes, and Public Health England have supported this by publishing mouth care guidance for patients with COVID-19 (31).

This is the first study to quantify the effect of periodontal disease on risk of COVID-19 infection and related outcomes. As such, there are several strengths to our study. First of all, this is the largest study to date to quantify the association between periodontal disease and COVID-19. Secondly, the utilization of UK Biobank, a national resource with high quality measures linked to COVID-19 test results enabled our quantitative analysis of association between periodontal disease and COVID-19 outcomes. Finally, our study demonstrates robust results using both imputed and complete cases.

Our study also has some limitations. Delayed information of hospital admission and mortality (updated until June 2020) limited the number of study cases, and has been recognized as an avenue for further investigation. The use of self-reported oral health indicators as a surrogate for signs of periodontal disease could introduce bias, as research suggests self-reported periodontal disease prevalence is underestimated in populations (32, 33). We are also aware that loose teeth, and painful or bleeding gums could result from trauma or endodontic diseases, however, there is evidence that the validity of a periodontal disease diagnosis is not compromised when self-reported responses of bleeding gums and loose teeth are utilized (18). In addition, the dataset held no information regarding any periodontal treatment that the participants had received in the time between reporting the oral measures and receiving the results of the COVID-19 test. Given that periodontal therapy can reverse or prevent disease activity, participants may have experienced changes to periodontal disease status between self-report at UK Biobank assessment center and undertaking a COVID-19 test. Furthermore, the oral measures (painful gum, bleeding gum and loose teeth) in this study may be due to oral dysbiosis but this causal association was not measurable within the UK Biobank dataset. Lastly, as the pandemic continues to take hold, the study follow up time for hospital admission and mortality was relatively short (until May and June 2020); the numbers of COVID-19 positive cases may have been underreported during the early stages of the pandemic.

Suggestions for future research include: (1) Using updated information of additional COVID-19 test results with longer follow up in order to acquire more precise estimates of the effect of periodontal disease on risk of COVID-19 infection and associated outcomes. (2) Utilization of linked dental and hospital records in future analyses. This could provide more accurate and recent information on oral health status in comparison to the self-reported periodontal disease responses at UK Biobank assessment centers and account for any change in oral health status following dental treatment.

## Conclusions

Our study demonstrated an increased risk of mortality following COVID-19 infection in people with periodontal disease. The findings suggest that while periodontal disease might not increase risk of COVID-19 infection directly, it may be associated with COVID-19 pathology and increase the risk of death. This indicates the importance of good oral hygiene and periodontal disease management, particularly while dental services are working below their pre-COVID-19 capacity levels.

## Data Availability Statement

The original contributions presented in the study are included in the article/Supplementary Materials, further inquiries can be directed to the corresponding author/s.

## Author Contributions

JW and JK contributed to study design and data acquisition and interpretation of the results. HL contributed to data analysis and interpretation and drafted the manuscript. SW contributed to interpretation of the results. All authors critically revised the manuscript. All authors contributed to the article and approved the submitted version.

## Conflict of Interest

The authors declare that the research was conducted in the absence of any commercial or financial relationships that could be construed as a potential conflict of interest.
