# Supplementary material for: The Impact of Periodontal Disease on Hospital Admission and Mortality During COVID-19 Pandemic
Source: Front Med (Lausanne). 2020 Nov 23;7:604980. doi: 10.3389/fmed.2020.604980 (PMC7719810; doi:10.3389/fmed.2020.604980)
Supplement: Supplementary file 1 [file Data_Sheet_1.docx]

Supplementary Material.

# Supplementary Tables

**Supplementary Table 1 Sensitivity analysis demonstrating association between oral health indicators and risk of COVID-19 infection without imputed data.**

|  | **Oral health status, OR (95% CI)** | |  |  |
| --- | --- | --- | --- | --- |
|  | **Normal** | **Painful Gums** | **Bleeding Gums** | **Loose Teeth** |
| **Crude OR (95% CI)** | **1 (ref)** | 1.12 (0.82-1.51) | 1.09 (0.77-1.54) | 0.85 (0.57-1.22) |
| **Adjusted OR (95% CI)** | **1 (ref)** | 1.13 (0.79-1.59) | 1.31 (0.88-1.97) | 0.69 (0.42-1.05) |

**Key:** confidence interval (CI), odds ratio (OR), reference value (ref),

***** Adjusted by age at test, sex, ethnicity, average total household income, BMI, systolic and diastolic blood pressure, history of smoking, history of previous conditions including: cancer, hypertension, angina, cardiac arrest, diabetes, myocardial infarction, stroke, peripheral artery disease, heart failure, atrial fibrillation and respiratory disease.

**Supplementary Table 3 Sensitivity analysis demonstrating association between oral health indicators and hospital admission and mortality for participants with COVID-19 infection without imputed data.**

|  | **Crude OR (95% CI) for oral health status** | | | | **Adjusted OR (95% CI) for oral health status** | | | |
| --- | --- | --- | --- | --- | --- | --- | --- | --- |
|  | Normal | Painful Gums | Bleeding Gums | Loose Teeth | Normal | Painful Gums | Bleeding Gums | Loose Teeth |
| **Hospital admission** | **1 (ref)** | 0.98 (0.35-3.62) | 0.80 (0.16-2.88) | 1.23 (0.34-9.23) | **1 (ref)** | 1.01 (0.25-4.92) | 0.84 (0.12-4.69) | 0.31 (0.05-3.32) |
| **Mortality** | **1 (ref)** | 1.60 (0.91-2.82) | 1.54 (0.81-3.01) | 2.03 (0.95-4.18) | **1 (ref)** | 1.77 (0.89-3.54) | 1.68 (0.74-3.95) | 2.62 (0.98-6.81) |

**Key:** confidence interval (CI), odds ratio (OR), reference value (ref),

***** Adjusted by age at test, sex, ethnicity, average total household income, BMI, systolic and diastolic blood pressure, history of smoking, history of previous conditions including: cancer, hypertension, angina, cardiac arrest, diabetes, myocardial infarction, stroke, peripheral artery disease, heart failure, atrial fibrillation and respiratory disease.
